# Retraction: Impact of nutrient warning labels on choice of ultra-processed food and drinks high in sugar, sodium, and saturated fat in Colombia: A randomized controlled trial

**DOI:** 10.1371/journal.pone.0303781

**Published:** 2024-06-10

**Authors:** Mercedes Mora-Plazas, Isabella Carolyn Aida Higgins, Luis Fernando Gomez, Marissa Hall, Maria Fernanda Parra, Maxime Bercholz, Nandita Murukutla, Lindsey Smith Taillie

After this article [1] was published, the authors notified the journal about errors in the calculation of the nutritional information used in the mock front-of-package labels used in this study.

The specific errors are:

- The calories and sugars reported for the no-sugar added fruit drink product in Table 1 are incorrect, resulting in an incorrect % of GDA value on the mock GDA label used in the study. The calorie value should have read 15 kcal, 1% of GDA, instead of 33.8 kcal, 2% of GDA. In addition, the sugars value should have read 3g, 3% of GDA, instead of 15.8g, 18% of GDA.
- The % of GDA value for sodium reported in Table 1 and used on the mock GDA labels are incorrect for all products, because the % of GDA was inadvertently calculated using the daily threshold for salt (6g) instead of sodium (2.4g) in each case.
- The Nutri-score values provided for the fruit drink products are incorrect, such that the “no-sugar added fruit drink” and the “fruit drink” were erroneously shown as having the same Nutri-Score value on the Nutri-Score label (both scored a B). The correct scores based on the mock product nutritional profiles are C and E, respectively. This error occurred because the fruit drink values were inadvertently calculated using a Nutri-score algorithm for foods rather than beverages.

In light of these errors, which impact the reliability and validity of the results, the authors retract the article. A replication study with an identical study protocol and sampling procedure with updated corrected nutrition labels has been published [2].

All authors agreed with the retraction.
